# Addressing disparities in European Social Sciences & Humanities research on climate, energy and mobility: insights from a Call for Evidence survey and analysis workshops on the challenges and opportunities of working in Southern and Central & Eastern Europe

**DOI:** 10.12688/openreseurope.16237.1

**Published:** 2023-09-18

**Authors:** Chris Foulds, Ami Crowther, Alevgul H. Sorman, Violeta Cabello, Dóra Bálint, Gergely Tagai, Viktor Varjú, Rosie Robison, Ester Galende Sánchez, Kristina Zindulková

**Affiliations:** 1 Global Sustainability Institute, Anglia Ruskin University, Cambridge, England, CB1 1PT, UK; 2 Basque Center for Climate Change, Bilbao, Basque Country, 48940, Spain; 3 Ikerbasque, Bilbao, Basque Country, 48009, Spain; 4 Institute for Regional Studies, Centre for Economic and Regional Studies, Pécs, 7621, Hungary

**Keywords:** Research geographies, Fragmentation, Research and innovation policy, Universities, Career pathways, Funding, Mixed methods.

## Abstract

Despite the efforts of the EU, disparities remain in terms of the participation of Social Sciences and Humanities (SSH) researchers from both Southern and Central & Eastern Europe in research collaborations, as compared to Northern and Western European scholars. To better understand these disparities, the EU Horizon Europe SSH CENTRE project ran a Call for Evidence over December 2022 to March 2023. Specifically, respondents were asked about the challenges they faced in conducting SSH research on climate, energy and/or mobility, as well as the ways in which these challenges could be addressed. The Call’s online survey was focused on maximising diversity, and it gathered views and experiences of 137 Southern and Central & Eastern European SSH researchers. The sample was balanced across genders (71 men, 66 women) and the three main themes (82 energy, 88 climate, 53 mobility), and included at least one respondent from each of the 27 target countries. The highest numbers of respondents were from Hungary (19) and Spain (21).

To ensure that interpretation and analysis of the data was grounded in regional contexts, we ran two parallel analysis workshops hosted in a hybrid format (combining online and in-person participants): one in Pécs for Central & Eastern European SSH researchers (34 participants); and one in Bilbao for Southern European SSH researchers (26 participants). These workshops focused on discussing the relationship between SSH-STEM disciplines, analysing the institutional contexts, and discussing the implications for domestic and EU research funding relations. During the workshops, data collected through the survey were collectively analysed and the most important reflections were gathered into a common structure of ‘Challenges’ and ‘Ways forward’. Key messages from the workshop are being distilled into a Position Statement that focuses on the common elements while also emphasising possible differences between Southern and Central & Eastern Europe.

## Plain language summary

Research on people and society (i.e. the Social Sciences and Humanities [SSH]) is essential if policymakers and practitioners are to deliver the changes that are urgently needed to address sustainability challenges. Yet, in comparison to the Technical and Natural Sciences (Science, Technology, Engineering, and Mathematics [STEM] disciplines) there are fewer opportunities for SSH research to contribute towards solutions. We would argue that this imbalance is exacerbated within Southern and also Central & Eastern European geographies, which have too often been in the shadow of the more dominant Northern and Western European geographies. For example, it has been shown that most of the funding, project consortia, policy advice, etc. opportunities (and thus prestige) are often associated with Northern and Western universities, or at least those that are closer to the Brussels policy bubble.

The ‘Social Sciences and Humanities for Climate, Energy aNd Transport Research Excellence’ (SSH CENTRE) project undertook a Call for Evidence to obtain the views and experiences of SSH researchers in Southern and Central & Eastern Europe. Insights from the Call for Evidence survey are being developed into a Position Statement that will be submitted to the European Commission with recommendations for how the geographical imbalance mentioned previously can best be addressed within SSH research and innovation policy making. This Data Note details the Call for Evidence’s online survey approach, including what questions were asked, why we asked them, and who responded. We also present our analytical approach which involved using these survey data as a prompt for two regional workshop discussions (involving participation of regional researchers only), which further deepened our understanding of the steps necessary to tackling these challenges.

## Introduction

It is increasingly acknowledged that Social Sciences and Humanities (SSH) research insights and evidence are essential if societies are to better understand the implications of, as well as influences behind, sustainability challenges ( Foulds & Robison, 2018; Ingeborgrud *et al*., 2020; Sovacool *et al*., 2015). It is in these contexts that dialogue has begun between research and innovation policy actors and SSH researchers, on how institutions need to evolve to help fulfil SSH’s unfulfilled potential ( *e.g.* Foulds *et al*., 2022; Krupnik *et al*., 2022; Ryghaug *et al*., 2023; Robison & Foulds, 2021).

It is certainly a positive sign that SSH is being taken more seriously and that key institutions ( *e.g.* European Commission) are starting to do more to establish and deliver upon SSH commitments in research and innovation programmes. However, evidence shows that the experiences of such programmes vary, and thus the expectations and distributional effects are not equal ( Royston & Foulds, 2021; Silvast & Foulds, 2022). This Note focuses on a particular issue in this respect, namely: current SSH research environments (on *e.g.* climate, energy and mobility) are dominated by scholars and institutions situated in Northern (N) and Western (W) Europe, with researchers from Southern and Central & Eastern Europe being less represented. There is thus a need to better balance research opportunities and participation across these different regions of Europe.

Reflecting calls to address such fragmentation issues, the Horizon Europe ‘Social Sciences and Humanities for Climate, Energy aNd Transport Research Excellence’ (SSH CENTRE) project ^1^ is writing a Position Statement that will be shared with the European Commission (Directorate-General for Research & Innovation). The Position Statement will focus on how SSH in Southern and Central & Eastern Europe can be better supported, with particular focus placed on supporting research focused on EU climate, energy and mobility policy.

The Position Statement will draw upon insights obtained through: (1) an online survey that focused on the experience of researchers within Southern and Central & Eastern Europe; and (2) two evaluation workshops (one in Bilbao, Spain; one in Pécs, Hungary) which supported the analysis and validation of the survey, as participants discussed challenges and ways forward for SSH within their respective geographies. This Data Note documents these data collection and analysis processes.

This Data Note is structured as follows: first, we outline and justify the methods that were used to collect insights on challenges, institutional contexts and funding relations. We then briefly set out the analysis undertaken and the process through which the survey data informed workshop discussions and the subsequent write-up of a Position Statement. The Data Note ends with a reflection on ethical considerations.

## Methods

### Research instrument selection

Responses to the Call for Evidence were collected through a web-based survey, using the Limesurvey software ^2^. The focus of the survey was on breadth and thus exposure to a variety of perspectives; in-depth insight was not the purpose of this survey. The use of online surveys, rather than other in-person methods, was fundamental in enabling access to the wide range of Southern and Central & Eastern European SSH communities, helping to capture a diversity of views, experiences and perspectives ( Wyatt, 2000).

### Research instrument design

***Country selection.*** As the Call for Evidence is part of a Horizon Europe project that develops understandings of how to better incorporate SSH insights into EU policy decisions, our starting point for countries to consider was: the 27 EU Member States; 16 non-EU Member States that are associated with Horizon Europe ^3^; plus the UK, which is seeking association post-Brexit and is currently participating in Horizon Europe *via* its UKRI guarantee. We also excluded countries that have recently had their EU research and innovation partnerships revoked ( *e.g.* Russia, Belarus).

Next, given our Call for Evidence’s focus on researchers with experience working (or who have received research training) in Southern or Central & Eastern Europe, we sought to establish which of these countries would be included within the scope of our survey. However, as there is no universally accepted framework for the regional grouping of European countries ( *i.e.* which are Southern or Central & Eastern), we developed our own categorisation system. Our final categorisation is available at Varjú *et al*. (2023), with 27 countries deemed to be eligible; 14 in Southern and 13 in Central & Eastern Europe.

In developing our regional categorisations, we studied existing categorisations for Southern and Central & Eastern Europe countries ( *e.g.* Central Intelligence Agency factbook ^4^, United Nations ^5^, University of Central Florida libraries ^6^) to identify commonalities in how different countries were treated. The variation across these existing categorisations highlighted the difficulty of developing these categories. For example, some countries can be considered as either Southern or Central & Eastern depending on the categorisation used ( *e.g.* Slovenia, Bulgaria). Furthermore, in some cases, we also observed that, for example, the category Central Europe did not exist, only Eastern Europe.

***The survey sample.*** The target group to complete the Call for Evidence survey was SSH researchers, although researchers with a STEM background were also able to respond. Recruitment occurred predominantly through a snowball sampling approach as the survey was shared through the SSH CENTRE consortium partners’ research networks. The aim was to obtain insights and perspectives from researchers across Southern and Central & Eastern Europe with a diverse range of characteristics ( *e.g.* gender, career stage, and SSH disciplines), rather than aim to achieve a statistically representative sample. As such, the snowball sampling approach was complemented by using publicly available databases of researchers ( *e.g.* Researchgate) and authors in relevant SSH journals ( *e.g.* Journal of Transport Geography, Sociology) to help identify individuals, which could plug gaps in our sample ( *e.g.* countries that at one time we had no responses for).

A total of 137 survey responses were collected between 16 December 2022 and 28 March 2023. A summary of the respondents’ main characteristics is available in Table 1.

**Table 1.** Main characteristics of Call for Evidence survey respondents.

	Southern European respondents (n=68)	Central & Eastern European respondents (n=69)	All respondents (n=137)
Country breakdown *	Spain (21) Italy (13) Portugal (6) Greece (5) Israel (5) Turkey (4) North Macedonia (3) Albania (2) Cyprus (2) Malta (2) Serbia (2) Bosnia and Herzegovina (1) Kosovo (1) Montenegro (1)	Hungary (19) Slovenia (9) Poland (8) Czechia (7) Estonia (6) Romania (6) Ukraine (3) Bulgaria (2) Croatia (2) Latvia (2) Republic of Moldova (2) Slovakia (2) Lithuania (1)	Spain (21) Hungary (19) Italy (13) Slovenia (9) Poland (8) Czechia (7) Estonia (6) Portugal (6) Romania (6) Greece (5) Israel (5) Turkey (4) North Macedonia (3) Ukraine (3) Albania (2) Bulgaria (2) Croatia (2) Cyprus (2) Latvia (2) Malta (2) Republic of Moldova (2) Serbia (2) Slovakia (2) Bosnia and Herzegovina (1) Kosovo (1) Lithuania (1) Montenegro (1)
Broad disciplinary research field	SSH (51) STEM (4) SSH-STEM interface (13)	SSH (53) STEM (4) SSH-STEM interface (12)	SSH (104) STEM (8) SSH-STEM interface (25)
Social Sciences and Humanities (SSH) discipline(s) **	Economics (9) Human Geography (5) Environmental Social Science (7) Politics (5) Sociology (6) Psychology (5) Business (2) Planning (2) History (1) Science and Tech Studies (2) Social Policy (1) Communication Studies (2) Development (1) Education (2) Law (2) Social Anthropology (2) Philosophy (1)	Economics (11) Human Geography (11) Environmental Social Science (7) Politics (7) Sociology (2) Psychology (1) Business (2) Planning (2) History (3) Science and Tech Studies (1) Social Policy (2) Development (1) Education (2) Law (2)	Economics (20) Human Geography (16) Environmental Social Science (14) Politics (12) Sociology (8) Psychology (6) Business (4) Planning (4) History (4) Science and Tech Studies (3) Social Policy (3) Communication Studies (2) Development (2) Education (2) Law (4) Social Anthropology (2) Philosophy (1)
Sustainability research area(s) **	Climate (41) Energy (42) Mobility (30)	Climate (47) Energy (39) Mobility (23)	Climate (88) Energy (82) Mobility (53)
Gender ***	Man (37) Woman (31) Prefer to self-describe (0)	Man (34) Woman (35) Prefer to self-describe (0)	Man (71) Woman (66) Prefer to self-describe (0)
Career stage	Current postgraduate student (2) Current postgraduate researcher (4) Early Career Researcher (14) Established Researcher (17) Senior Researcher (31)	Current postgraduate student (2) Current postgraduate researcher (5) Early Career Researcher (16) Established Researcher (24) Senior Researcher (22)	Current postgraduate student (4) Current postgraduate researcher (9) Early Career Researcher (30) Established Researcher (41) Senior Researcher (53)

* Based on the answer to the following survey question: “I am answering these questions in relation to the following country” ( *i.e.* it was not based on their nationality or where they were currently living). ** Respondents could tick more than one answer. *** Their gender was not assumed. A survey question asked the respondents to explicitly self-assign their genders.

The number of responses is well-balanced between Southern (68 responses) and Central & Eastern (69) Europe countries. Importantly, we received at least one response from every one of the 27 eligible countries. The highest number of respondents were from Spain (21) and Hungary (19), where the two analysis and validation workshops on the survey responses took place. Responses were also fairly balanced in terms of gender representation (71 men, 66 women).

The majority of respondents held PhD degrees, with responses from senior researchers (54), established researchers (31) and early career researchers (30) dominating the responses. Among Southern European respondents, there was a higher share of those who identified themselves as senior researchers (31 out of 69), while in the case of Central & Eastern Europe a similar number of respondents represented senior (22) and established researchers (24).

The research domain of respondents was predominantly SSH (104 out of 137), although 25 respondents identified as interdisciplinary researchers (i.e. spanning both SSH and STEM to various extents). The number of responses from purely STEM researchers was low (8). Across the Southern and Central & Eastern European respondents, there were similar levels of responses from SSH, STEM and interdisciplinary researchers. Considering the disciplines covered in the survey, the most represented SSH disciplines were Economics (20), Human Geography (16), Environmental Social Science (14), and Politics (12). At least one response was also submitted from each of the disciplines of Communication Studies, Development, Education, Law, Social Anthropology, and Philosophy.

***Constructing survey questions.*** The Call for Evidence survey was structured around four sections: (1) the nature of collaboration between SSH and STEM; (2) the position of SSH in the countries’ institutional systems (compared to STEM); (3) the position of SSH in funding opportunities; and (4) ease or difficulties in joining EU research projects/funding. The survey included both qualitative and quantitative questions, with the combination of these questions enabling insights that both provided an overall picture of respondents and their characteristics (including *e.g.* age, gender, career stage), as well as richer detail related to their views and experiences ( Esturo *et al*., 2023). The survey questions provided respondents with the opportunity to reflect on how they could be supported during collaborative research with STEM, in institutional settings, and also in terms of funding opportunities. The full set of survey questions are available at Varjú *et al*. (2023).

The questions were developed through an iterative process. The survey was initially piloted (16 November – 13 December 2022) with selected SSH CENTRE consortium members based in Southern and Central & Eastern geographies, particularly with a focus on completion duration and comprehension of survey questions. Several rounds of iterations helped improve question styles, unique country identifiers, as well as how to cater for a range of disciplinary fields.

## Survey analysis, workshop validation procedures and identification of recommendations

The survey responses were discussed and validated during two regional hybrid workshops, en route to recommendations being produced for submission to the European Commission (in the form of a Position Statement with 12 recommendations). The three data analysis and validation steps are now detailed.

### Step 1 - Interpreting survey responses

Step 1 of analysing and validating the gathered 137 survey responses involved two analyses. First, a thematic analysis was conducted on the qualitative survey data, particularly focusing on the open questions ( *i.e.* related to experiences and perspectives). Second, basic descriptive statistics were performed on the quantitative data, primarily concerning the demographic information of the survey respondents.

Both of these analyses aimed to identify common issues across the responses from Southern and Central & Eastern Europe, as well as within each region individually.

The outcomes of these analyses yielded two main results. Firstly, several priority discussion themes inductively emerged, supported by relevant quotations, which served as focal points for Step 2. For Southern Europe, three priority themes were identified: (i) SSH visibility and SSH-STEM relations; (ii) institutional support; and (iii) funding. For Central & Eastern Europe, a similar three were also identified: (i) roles and position of SSH in Central & Eastern European countries in energy, climate and mobility; (ii) funding; and (iii) solutions. Secondly, information about the respondents' sample and background was provided, offering valuable context for understanding the identified priority themes and quotations.

### Step 2 - Collective data analysis and discussion via workshops

Two hybrid analysis workshops were held consecutively: one in Southern Europe in Bilbao ^7^, and one in Central & Eastern Europe in Pécs ^8^. Participation in the workshop was by invitation, to help ensure diverse geographical and disciplinary representation, as well as a gender balance. Each workshop participant had experience of undertaking research within Southern or Central & Eastern Europe. They also either identified as an SSH researcher or were supportive of engaging with SSH research. Before the workshop, all participants needed to have completed the Call for Evidence survey too.

The majority of the workshops’ 60 participants ^9^ were identified through SSH CENTRE consortium contacts or searches of relevant research institutions in Southern and Central & Eastern Europe. When wanting to address particular gaps in either geography or discipline, relevant survey respondents (who consented to being contacted about the workshop) were invited. Collectively, the participants of the workshops had experience working in at least 18 SSH research disciplines, as well as research environments in 23 different Southern and Central & Eastern European countries.

Inspired by previous experiences on participatory analysis of mixed quantitative and qualitative data ( *e.g.* Di Felice *et al*., 2021), the workshops were a means of situating the Call for Evidence and collectively analysing and interpreting answers with a wider invited audience of Southern or Central & Eastern European researchers. Participants of both workshops therefore were also invited to be co-authors of the Position Statement, as the conclusions and recommendations were co-designed (see ‘Step 3’ section below). Co-hosting the workshops with partners beyond the project’s consortium also helped provide the means to situate the debate with researchers from across the region, thereby bringing in diverse perspectives from research/action spaces on-the-ground.

Both workshops followed a similar structure: following a presentation of the survey respondent sample descriptive statistics, we interrogated and began with the primary themes identified in Step 1. We presented the themes in-depth - including *e.g.* challenges, tensions, opportunities, perceptions, unanswered questions, *etc.* - and evidenced these with a range of quotations that were sourced from respondents in their region ( *i.e.* either Southern or Central & Eastern Europe). It was important that the workshop’s analysis and validation discussion remained at the regional level, thereby ensuring that local contexts could be better accounted for.

Then, smaller breakout groups (of five-eight people) were formed to provide the space for participants to reflect (via facilitated negotiation) on the themes presented. These reflections were summarised in writing by facilitators. Regrouping at plenary sessions then enabled knowledge exchange from the breakout groups as well as provided a space for collective deliberation and aligning priorities.

The outcomes of Step 2 were thus in-depth, detailed facilitator notes that synthesised and validated priority issues, to better support SSH in Southern or Central & Eastern Europe. The personal nature of these in-depth discussions (relating to *e.g.* career experiences) meant that these data could not be meaningfully anonymised and thus were not publicly shared.

### Step 3 - Identifying recommendations in the form of a Position Statement

During Step 2, in the plenary sessions, there was an emphasis on (i) noting points of divergence and convergence across the workshop participants, and (ii) discussing overarching key messages and ways in which recommendation statements could potentially be narrated. The two sets of written outputs (together with the original survey response and analysis) then informed the development of a draft Position Statement that captured challenges experienced by SSH researchers in Southern and Central & Eastern Europe, as well as suggested actions that funders and research institutions in particular can take, through 12 recommendations. This draft Position Statement was circulated to workshop participants for feedback and final refinement in May 2023. The Position Statement will be shared with the European Commission (Directorate-General for Research & Innovation).

## Ethics and consent

The survey, and its analysis method, was approved by the School Research Ethics Panel located within Anglia Ruskin University’s Global Sustainability Institute (reference number of ETH2223-0756; approval date of 11 November 2022). This process ensured that the questions asked and data collected: complied with the university’s ethical requirements; ensured written informed consent; and was compliant with the EU General Data Protection Regulation (GDPR).

The front matter of the survey outlined (1) the purpose of the survey; (2) the type of data that would be collected ( *e.g.* Name/Contact Details, Age, Gender, Experiences); (3) how this data would be used; and (4) who would have access to the data. In order to proceed to the survey itself, survey respondents were required to tick boxes related to consent statements, with this ensuring informed consent was obtained. These statements included agreeing to participate in the survey, understanding who would be able to access the data (complying with GDPR), and understanding that the data would be anonymised. The survey was designed to only include questions that provided relevant and required data, thus adhering to the principle of data minimisation too.

The anonymisation of the data occurred in two phases. The first phase included going through the qualitative responses to open-ended questions and removing all references to places, projects and organisations that could be used to identify an individual respondent. The second phase of anonymisation involved separating the demographic data collected ( *e.g.* age, gender, career stage) from the other survey responses that focused on respondent experiences. The demographic data was then randomised, so that there would be no cross-referencing ( *i.e.* between the respondent demographic and the respondent experiences questions), thus reducing the likelihood of individual respondents being identifiable.

## Data Availability

All data and materials are available on Zenodo. DOI: https://doi.org/10.5281/zenodo.8033610 **SSH CENTRE: T1.3.1 Call for evidence survey data file**: This project contains the following data: ○  The full, anonymised version of the survey export file (xlsx) from Limesurvey, including consent statements and all participant responses. DOI: https://doi.org/10.5281/zenodo.8080979 **SSH CENTRE T1.3.1 Call for Evidence survey extended data**: This project contains the following data: ○  The contents of the survey ( *i.e.* survey front matter and all survey questions), as well as the list of selected countries that were classified as Southern European or Central & Eastern European for the purposes of this research project. DOI: https://doi.org/10.5281/zenodo.8033576 **SSH CENTRE: T1.3.2 SE, CEE Workshop information**: This project contains the following data: ○  The agendas and consent form used in the two hybrid workshops, as part of collectively analysing the Call for Evidence survey data/findings and distilling relevant recommendations. Data are available under the terms of the Creative Commons Attribution 4.0 International licence (CC-BY 4.0).
