# Supplementary material for: Addressing disparities in European Social Sciences & Humanities research on climate, energy and mobility: insights from a Call for Evidence survey and analysis workshops on the challenges and opportunities of working in Southern and Central & Eastern Europe
Source: Open Res Eur. 2023 Sep 18;3:150. [Version 1] doi: 10.12688/openreseurope.16237.1 (PMC10646341; doi:10.12688/openreseurope.16237.1)
Supplement: Peer review history

# Reviewer report: Reviewer response for version 1

**DOI:** 10.21956/openreseurope.17531.r36051

**Published:** 2023-11-14

**Authors:** Federica Rotondo

**Affiliations:** 1 Politecnico di Milano, Milan, Italy

**Summary:**

 The “data note” focuses on the disparities in terms of research opportunities and participation for SSH and STEM researchers to contribute to projects addressing sustainability challenges. Particular attention is given to the imbalance between Southern and Central & Eastern European countries (less represented) and Northern and Western European countries (highly predominant).

 The work develops a three steps approach. At first an on-line survey is elaborated and diffused to better understand the experience of researchers in Southern and Central & Eastern European countries. After that, two hybrid workshops are organised to discuss the survey results and to outline possibile ways forward for SSH researchers based in different locations. Finally insights of the activities lead towards the elaboration of a Position Statement that will be shared with the European Commission, including some recommendation to address the geographical imbalance of SSH research and to support SSH researchers of central/eastern/southern European countries

**General comments and specific issues:**

 Overall, the contribution is well structured and presents original features. Some improvements can be made in the direction of better specifying methods and materials at different steps of your work. Moreover the anonymous version of the survey outcomes is not accesible to the author of this review. Below are some useful points for the implementation of the paper.

- About the sentence “it has been shown that most of the funding, project consortia, policy advice, etc. opportunities (and thus prestige) are often associated with Northern and Western universities, or at least those that are CLOSER to the Brussels policy bubble”.  Do you mean geographically closer? Or, for example, politically closer? You should either detail or reframe the sentence.
- About the country selection: to better clarify your categorisation of countries you could specify the criteria you adopted to categorise a country as part of southern/central/Eastern Europe. For example: by looking at the pdf "country groups_3" (https://zenodo.org/records/8080979) it seems that you included Czech Republic, Slovakia, Hungary and Slovenia but not Austria. The reason is not fully clear.
- On the survey sample: to better understand the snowball sampling phase it would be helpful to explicit what are the disciplines of researchers involved in the SSH CENTRE consortium partners and to report what are the disciplines of the 137 respondents.
- On step 1: the priority themes that emerged from the interpretation of survey responses could be expanded. I would like to better understand what the respondents refer to by mentioning "SSH visibility" or "SSH-STEM relation" or what is the different claims about "funding" in Southern European and in Central & Eastern Europe.
- On step 2: you could specify, in a table or graph, what are the 18 SSH research disciplines and the 23 Southern and Central & Eastern European countries of the participants of the workshops. Since you pointed out that the discussion remained at the regional level, I think you should better explain what you mean by regional level.
- About step 2 outcomes: you could better specify how the discussions lead to the synthesis and validation of priority issues. After the discussion how the priority issues eventually change? Based on which criteria? Could you also better specify the role of facilitator?
- About step 3: it would be useful to understand the general structure of the draft position statements that circulated to workshop participants for feedback. How did you take into account the regional specificities?

Are sufficient details of methods and materials provided to allow replication by others?

Partly

Is the rationale for creating the dataset(s) clearly described?

Yes

Are the datasets clearly presented in a useable and accessible format?

Partly

Are the protocols appropriate and is the work technically sound?

Yes

Reviewer Expertise:

Urban planning, urban policy and design

I confirm that I have read this submission and believe that I have an appropriate level of expertise to confirm that it is of an acceptable scientific standard.

# Reviewer report: Reviewer response for version 1

**DOI:** 10.21956/openreseurope.17531.r35497

**Published:** 2023-11-14

**Authors:** Giulia Sonetti

**Affiliations:** 1 CENSE – Center for Environmental and Sustainability Research & CHANGE - Global Change and Sustainability Institute, NOVA School of Science and Technology, NOVA University Lisbon, Caparica, Portugal

The article provides a comprehensive overview of a survey conducted to understand the dynamics of SSH and STEM collaborations in Southern and Central & Eastern Europe. The rationale for creating the dataset is well-articulated, focusing on exploring the nature of collaboration, institutional positioning, funding opportunities, and ease of joining EU research projects.

 The methods employed for data collection and analysis are robust, involving a mixed-methods approach with qualitative and quantitative elements. The iterative development of survey questions and pilot testing adds to the credibility of the process. However, for replication purposes, the authors could provide more detailed information on the specific methodologies for thematic analysis and statistical analysis.

 While the dataset's composition is described in terms of the type of responses collected, information on the format and accessibility of the dataset for public use is somewhat lacking. To enhance the utility of this research, the authors should consider providing clear details on how the dataset is formatted and where it can be accessed.

 Overall, the study is well-conducted and provides valuable insights into SSH and STEM collaborations in the European context. To enhance its scientific soundness and replicability, minor improvements in detailing the analytical methods and clarifying the dataset's presentation are recommended.

Are sufficient details of methods and materials provided to allow replication by others?

Partly

Is the rationale for creating the dataset(s) clearly described?

Yes

Are the datasets clearly presented in a useable and accessible format?

Yes

Are the protocols appropriate and is the work technically sound?

Yes

Reviewer Expertise:

Transdisciplinarity, Sustainability Science

I confirm that I have read this submission and believe that I have an appropriate level of expertise to confirm that it is of an acceptable scientific standard.

# Reviewer report: Reviewer response for version 1

**DOI:** 10.21956/openreseurope.17531.r36049

**Published:** 2023-11-14

**Authors:** Thomas Berker

**Affiliations:** 1 Department of Interdisciplinary Studies of Culture, Norwegian University of Science and Technology, Trondheim, Norway

Thank you for the opportunity to try out this open peer review process, which was a new experience for me. That the review is openly accessible together with the article allows me to comment without forcing the authors to respond or alter their text. This is why I divide the following remarks between "methodological reflections" that may or may not be useful for the authors and the reader and recommendations for minor changes that in my view would improve the text.

Short summary:

 This data note describes and justifies the methodological choices made in a research project on the challenges and opportunities of working in Southern and Central/Eastern European SSH research on climate, energy and mobility. The empirical work consisted of an online survey (N=137) and two hybrid workshops (N=60).

Recommendations:

- The sampling strategy was nonprobabilistic and based on self-selection and snowball sampling. Both are pragmatic and effective but snowball sampling has the effect that networks of the people selected initially will dominate the sample. Effects of this should be discussed in the data note.
- Is it possible that in addition to snowball sampling, elements of purposive sampling and maybe also a weak form of quota sampling were employed? The latter is hinted at when the goal 'to achieve a diverse range of characteristics' (gender, career stage, disciplines) is mentioned (p.4). Did you do active work to fill specific quotas (e.g., of women) or am I misunderstanding this remark?
- Probably to broaden participation, a weak criterion for being able to talk about/for Southern/Eastern/Central European SSH was used: "some time studying at postgraduate level or working as a researcher". Question 19 [Q_2a]a asks whether respondents currently live in the country they are answering for, while the following questions are open for respondents that may only have had short stays there, potentially many years ago. What is the reason for including both types of questions? It does make a difference whether one is answering after having left the country or not, but was this difference used in the analysis? More information in the data note on these questions of definition would be useful.
- Why did you include STEM researchers in the sample? Surely not to compare experiences between STEM and SSH respondents (the STEM group is too tiny for this)?
- The relation between workshops and the survey remains somewhat unclear. First you did a thematic analysis of the survey results which produced quotes that could be used as 'focal points' for the workshops. Additionally, very broad priority themes (e.g., 'funding', 'visibility', 'STEM-SSH-relations') were identified that were discussed at the workshops. But the terms 'collective data analysis' and 'validation' hint at a stronger relation. The outcome here, 'in-depth, detailed facilitator notes' (that cannot be published) seems like a weak outcome of the 'collaborative analysis' and 'validation'. It might be advisable to avoid these terms or to define better what they mean in the context in which they are used here.

Methodological reflections:

 In any empirical study, how the object of inquiry is constructed should be carefully justified. The danger to create a method artifact, i.e. to construct objects that say more about the methodological choices than about anything else, is always real. The assumption that SSH researchers from Southern and from Central/Eastern Europe experience a set of common problems compared to their Western and Northern European colleagues makes only sense if one accepts the premise that they are a group - or maybe two groups - with common features and working under similar conditions. The authors acknowledge that Eastern, Central and Southern European are categories that are understood differently and may entail unclear boundaries and overlaps. The chosen solution - a catalogue of countries based on pragmatic, institutional and political reasoning - is good enough. However, what may be more problematic is that these broad categories include very different countries with widely different research landscapes, histories, economic contexts, etc. And then there are obviously disparities within countries and also within institutions and between different SSH disciplines. In this context it may be particularly problematic that the snowball sampling for the survey started with consortium members, i.e. researchers that are engaged in an ongoing collaboration with powerful European research nodes that - not accidentally - reside in Western Europe.

 Moreover, large parts of European SSH research were excluded from the empirical inquiry through the assumption that the English language is the lingua franca in all academic contexts, which is particularly untrue in SSH. The authors were aware of the danger that they may end up creating a research object, which may or may not say anything about SSH research in Southern and Central/Eastern Europe. The workshops in Hungary and Spain were explicitly framed as a way to validate and situate the debate in a specific region, e.g. by including local researchers that were not part of the consortium. Ideally, these workshops would have allowed to shed light on disparities in European SSH in their specific, multi-dimensional form (within institutions, between disciplines, between institutions, between regions, etc.). However, situated accounts like this would not be able to become position statements and generate input suitable for the European Commission. But they would avoid the temptation to 'see like a (transnational) state', which constructs its objects according to its priorities. The objects Southern and Central/Eastern Europe make a lot of sense in the context of European economic integration. Whether they are good objects for an empirical investigation of inequality in European research is another question - a question which is not answered by this data note.

Are sufficient details of methods and materials provided to allow replication by others?

Yes

Is the rationale for creating the dataset(s) clearly described?

Yes

Are the datasets clearly presented in a useable and accessible format?

Yes

Are the protocols appropriate and is the work technically sound?

Yes

Reviewer Expertise:

Science and technology studies, sociology

I confirm that I have read this submission and believe that I have an appropriate level of expertise to confirm that it is of an acceptable scientific standard.

# Reviewer report: Reviewer response for version 1

**DOI:** 10.21956/openreseurope.17531.r35499

**Published:** 2023-11-03

**Authors:** Osman Arrobbio

**Affiliations:** 1 Department of Humanities, Social Sciences and Cultural Industries, University of Parma, Parma, Italy

The EU Horizon Europe SSH CENTRE project aimed to understand and address disparities in the participation of Social Sciences and Humanities (SSH) researchers from different European regions in climate, energy, and mobility research. More precisely, this research project addresses the underrepresentation of SSH researchers from Southern and Central & Eastern Europe.

 The authors collected 137 responses from Southern and Central & Eastern Europe SSH researchers, conducted two regional workshops, and are preparing a Position Statement with recommendations for the European Commission to address these disparities.

 A web-based survey was used to collect responses from SSH researchers in Southern and Central & Eastern Europe.

 The survey and its analysis were approved by an ethics panel. Informed consent was obtained through a clear explanation of the survey's purpose and data handling, with active agreement from respondents. Data minimization principles were followed to collect only necessary data.

 Snowball sampling and publicly available databases were used for recruitment.

 The survey questions covered collaboration, institutional support, funding, and participation in EU projects.

 Data analysis and validation were done in three steps:

- Step 1 involved thematic analysis and descriptive statistics.
- Step 2 consisted of two regional workshops in Southern and Central & Eastern Europe to discuss the survey data.
- Step 3 focused on identifying recommendations for a Position Statement.

The workshops involved participants from various disciplines (mainly SSH) and countries from the investigated European regions, and the findings informed the development of the Position Statement.

 The author can find below some comments and suggestions (neither listed in order of importance nor appearance):

1. On the use of “European” or “Europe” (for example, in sentences like “[…] it gathered views and experiences of 137 Southern and Central & Eastern European SSH researchers”). Please consider that I am not a native English speaker, but I wonder whether the use of “Europe” would make clearer to the reader that those SSH researchers are from S and C&E Europe and may thus not necessarily have a S or C&E European nationality.
2. Through which channel(s) were people invited to participate to the survey? E.g. through personal contacts, through communications on websites?
3. “[…] those who identified themselves as senior researchers (31 out of 69) […]” Maybe it is 31 out of 68?
4. “[…] while in the case of Central & Eastern Europe a similar number of respondents represented senior (22) and established researchers (24).” Add “out of 69”?
5. “The research domain of respondents was predominantly SSH (104 out of 137)”. What about making clear that the research domain was self-reported by respondents?
6. I would make clear that the survey was in English.
7. “The highest number of respondents were from Spain (21) and Hungary (19) […]”. This does not completely correspond to what is written in one of the notes to Table 1, where it says that “Based on the answer to the following survey question: ‘I am answering these questions in relation to the following country’ (i.e. it was not based on their nationality or where they were currently living).” I suggest rephrasing it like this (or any other more elegant form): “The highest number of responses were related to Spain (21) and Hungary (19)”.
8. “At least one response was also submitted from each of the disciplines of Communication Studies, Development, Education, Law, Social Anthropology, and Philosophy”. If I have well understood, this list contains the least represented disciplines. However, from what I see in Table 1 there are some disciplines (Science and Tech Studies; Social Policy) that are less represented than Law. Please, check those figures in Table 1 and modify the list (in the reported sentence and/or the ordering of disciplines in Table 1).
9. Table 1. Section ‘Social Sciences and Humanities (SSH) discipline(s)’. Please, specify whether this question was posed only to those who self-defined as SSH researchers?
10. Table 1. Section ‘Sustainability Research Areas’. Please check the figures related to Energy.
11. Table 1. I wonder if you could also add the age of respondents.
12. You listed the three priority themes for both regions. Are these priorities listed by importance? In case I would specify it.
13. “The majority of the workshops’ 60 participants were identified through SSH CENTRE consortium contacts […]”. Would it be possible to include the composition of the SSH CENTRE consortium (e.g., among the supplementary materials)?
14. Not an expert on this, but please consider if it is necessary/useful to remove some data (e.g. from open-ended answers) from the supplementary material. I mean, in order to decrease even more the possibility for respondents to be identifiable.
15. In the paragraph where you describe the workshops structure, I suggest you to say that the agendas can be found among the supplementary materials.
16. Workshops were held in a hybrid (online and in person) format. Did this create difficulties? If yes, how did you manage them?
17. Among the supplementary materials, I suggest you clean up the xlsx file a little bit to make it more quickly usable. For example, some respondents used “Czeck Republik” and others “Czekia”. Maybe you could decide on just one formulation. Also, some countries have two “versions” depending on whether respondents add or not a space after the name of the country. (e.g. the cases of Israel and Greece).

Are sufficient details of methods and materials provided to allow replication by others?

Partly

Is the rationale for creating the dataset(s) clearly described?

Yes

Are the datasets clearly presented in a useable and accessible format?

Partly

Are the protocols appropriate and is the work technically sound?

Yes

Reviewer Expertise:

Sociology of the environment. Sociology of energy.

I confirm that I have read this submission and believe that I have an appropriate level of expertise to confirm that it is of an acceptable scientific standard, however I have significant reservations, as outlined above.
